# A minimally invasive surgical approach for the treatment of piriformis syndrome: a case series

**DOI:** 10.1186/s41016-020-00189-y

**Published:** 2020-03-30

**Authors:** Elizabeth Hogan, Darshan Vora, Jonathan H. Sherman

**Affiliations:** 1 Department of Neurosurgery, the George Washington University, 2150 Pennsylvania Avenue, NWSuite 7-420, Washington, DC 20037 USA; 2 Medicine and Health Sciences, the George Washington University, Washington, DC USA

**Keywords:** Piriformis syndrome, Sciatic nerve, Minimally invasive, Neuromonitoring

## Abstract

**Background:**

Piriformis syndrome accounts for approximately 6% of patients who present with sciatic pain. There are many treatment options ranging from physical therapy, to trigger point injections, to surgical intervention. We discuss a surgical method that represents a minimally invasive technique for the treatment of piriformis syndrome.

**Methods:**

We describe a novel operative approach and technique for release of the piriformis muscle in the treatment of piriformis syndrome. Described are the preoperative planning, incision and approach, and technique for identifying and releasing the piriformis muscle.

**Results:**

Three patients were treated for piriformis syndrome using the described technique. Each patient displayed successful relief of their symptoms immediately following the surgical procedure and at delayed follow-up.

**Conclusion:**

Early experience with our method of piriformis release suggests that it is well suited for the treatment of piriformis syndrome. The novel integration of pre-operative trigger point localization coupled with intraoperative neuromonitoring allows effective pain relief with minimal morbidity.

## Background

Piriformis syndrome (PS) is a controversial and uncommon condition that is characterized by symptoms caused by irritation or compression of the sciatic nerve as it travels underneath the piriformis muscle just distal to the sciatic notch [1]. It is speculated that about 6–8% of sciatic nerve pain is due to PS [2, 3]. However, it is believed that PS is often under diagnosed because it is clinical diagnosis and often confused for other more common conditions such as lumbar disc herniation or facet arthropothy [4]. PS is characterized by buttock pain that radiates down the posterior aspect of the leg that is aggravated by sitting. Patients also report external tenderness near the greater sciatic notch [1].

The treatment of PS has traditionally been conservative in nature with noninvasive treatment including activity modifications, physical therapy, and use of anti-inflammatory medications, muscle relaxants, and/or neuropathic pain medications [1, 5]. When patients fail conservative management, the next step in treatment is typically localized injections of either corticosteroids or botulinum neurotoxin into the piriformis muscle [5]. Surgical treatment of PS is reserved for patients that are refractory to conservative management. Current surgical methods to treat PS include open or endoscopic decompression of the sciatic nerve by release of the piriformis muscle [6]. The purpose of this paper is to describe a new minimally invasive surgical technique and its operative nuances that were used to treat 3 patients with PS.

## Methods

A series of 3 patients were chosen to undergo this surgical technique for treatment of PS. They were treated between 2012 and 2016 by the senior author. The 3 patients selected had all failed conservative management and met the criteria for surgical intervention. Institutional review board (IRB) approval was not obtained because it was not required for case reports or case series at our institution. Our hospital IRB states that cases with 4 or less patients do not need to be reviewed. Additionally, all patients mentioned in the series were of the investigators’ own patients involved in direct patient care. Therefore, consent from the patients used in this study was not obtained because this study is exempt under 45-CFR-46.

Prior to surgical selection, the 3 chosen patients underwent a thorough examination and workup. Furthermore, they all had to attempt conservative medical treatment including steroid injections and physical therapy for at a minimum of 6 weeks. Given their presenting symptoms, the number one differential to rule out was lumbar radiculopathy. Therefore, they all underwent magnetic resonance imaging (MRI) of their lumbar spine, which were negative for any pathology that could explain their current symptomology. Additionally, the senior author of this paper, a board certified neurosurgeon who has been working in neurosurgery and with peripheral nerve disorders since 2003, examined and selected each patient for surgical intervention.

The first step for the surgical treatment of PS was to localize the trigger point in the pre-operative area and clearly mark it with a marking pen. This is the critical step in our new technique, because the trigger point was used as the entry point for a surgery. Applying firm point pressure in multiple areas around the anatomical location of the pisiforms muscle until the exact location is found that recreates the patient’s sciatic nerve pain and is how the trigger point is identified. Once in the operating room, the patients were placed under general anesthesia and positioned prone on the operating table. Then, a 3–4-cm incision was drawn with the trigger point as the midpoint of the incision along the direction of the piriformis muscle between the sacrum and the greater trochanter of the femur. The patients were then prepped and draped in a standard surgical fashion.

**Fig. 1:**
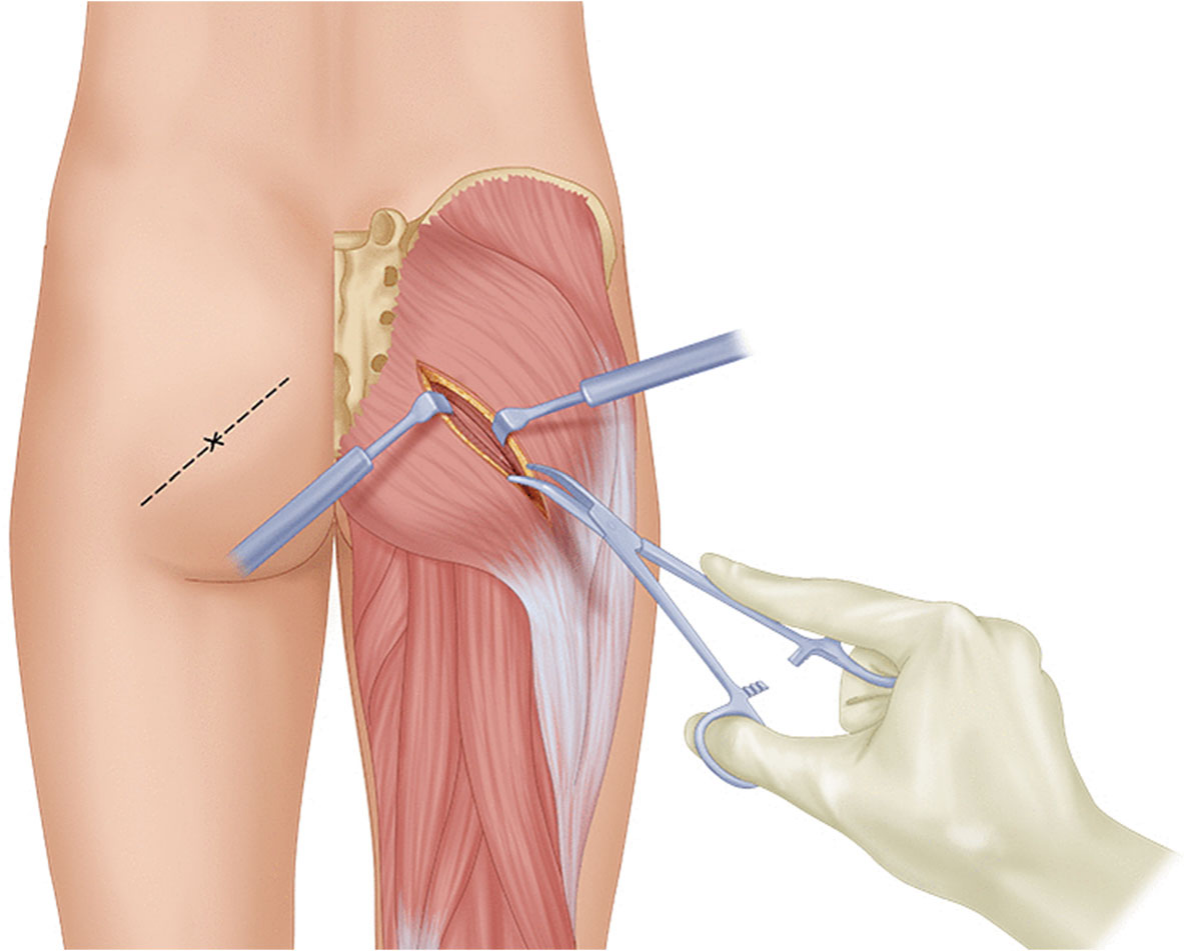
Illustration of displaying the incision line and initial dissection through the gluteus maximus. On the right side of the image, the incision line is displayed and center on the trigger point identified in the pre-op area. The line is drawn along the direction of the piriformis muscle extending from the sacrum to the greater trochanter of the femur. On the right side of the image, the initial blunt dissection is displayed through the gluteus maximus

Following draping, 10 cc of 0.25% Marcaine with epinephrine was injected into the epidermis and dermis layer. Following incision with a 10 blade, a secondary knife and a monopolar were used to cut down through the subdermal fat layer until the fascia over the gluteus maximus was identified. An Adson-Beckman retractor was then placed. Subsequently, Metzenbaum scissors were used to cut the fascia over the gluteus maximus, and a tonsil dissector was used to bluntly dissect through the gluteal muscle until the inner fascia is identified (Fig. 1). The fat pad surrounding the sciatic nerve is then identified just distal to the overlying piriformis.

**Fig. 2:**
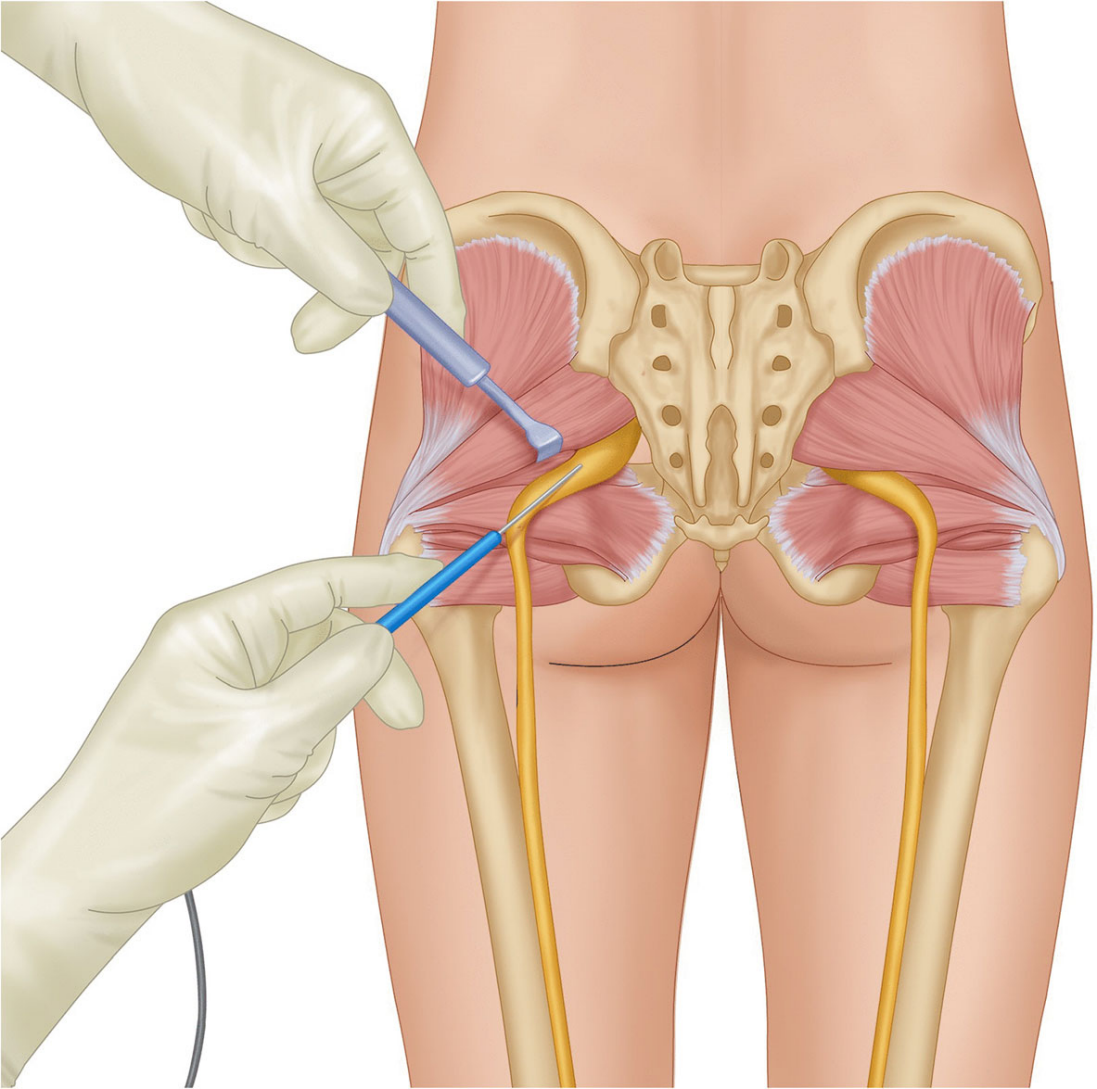
Illustration of displaying stimulation and identification of the sciatic nerve using a nerve stimulator. The piriformis muscle is elevated with a hand-held retractor in this process

**Fig. 3:**
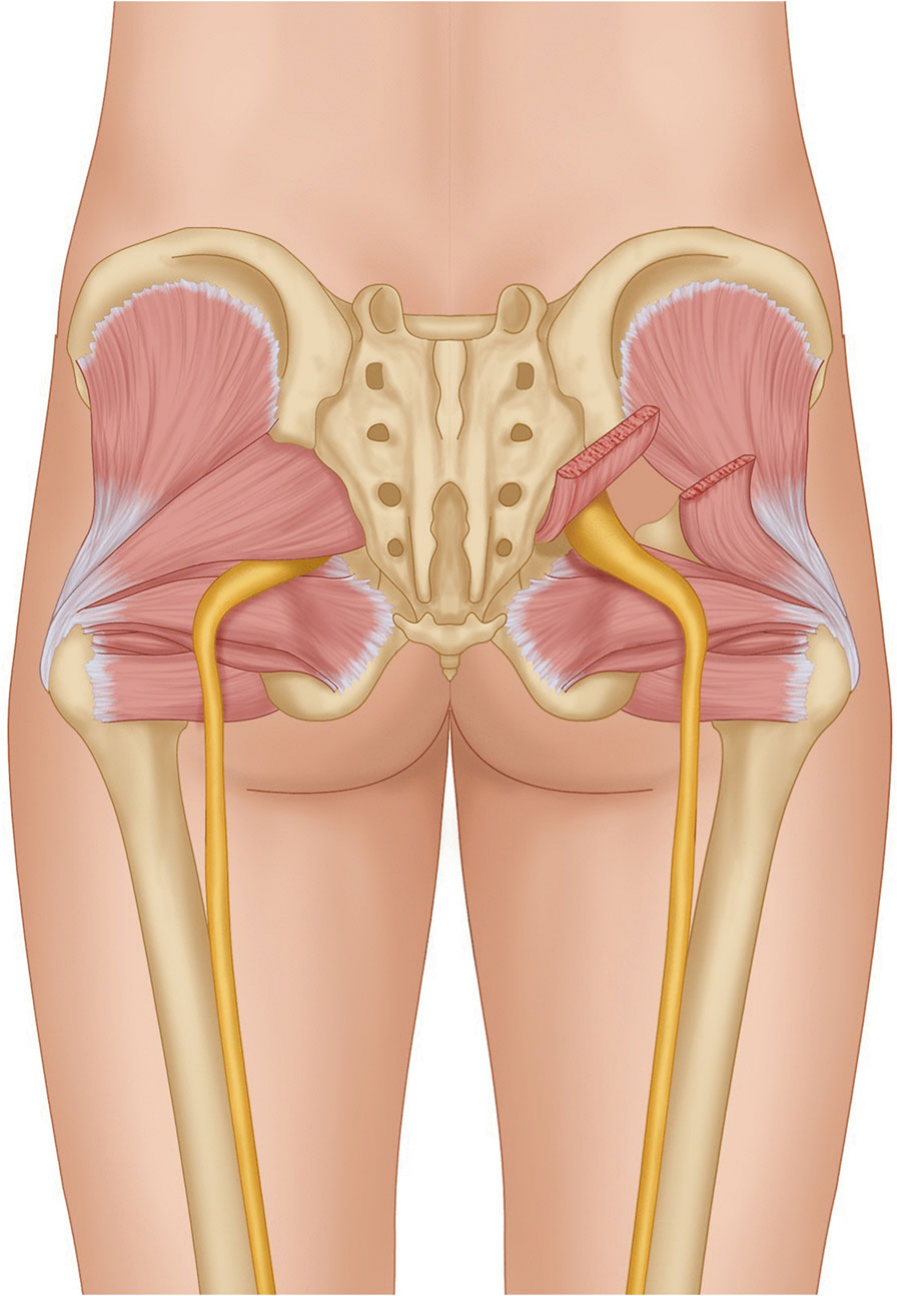
Illustration of displaying the release of the piriformis muscle. On the left side of the image, the sciatic nerve is displayed in its typical location below the piriformis muscle. On the right side of the image, the sciatic nerve is shown decompressed after cutting of the piriformis muscle

Once the fat pad is identified, the sciatic nerve is confirmed using electromyography (EMG) with stimulation at 0.5 mA producing muscle twitches in the distribution of the sciatic nerve. A hand-held retractor is used to elevate the piriformis muscle in this process (Fig. 2). After the nerve is identified with EMG, it is protected with a 1 × 1 paddie. At the base of the piriformis muscle, a vascular bundle is typically identified and coagulated. Monopolar electrocautery is then used to completely cut the piriformis muscle until the muscle is completely released (Fig. 3). A blunt dissector is then used to confirm that the sciatic nerve is completely decompressed.

The fascia overlying the gluteus maximus is then closed with interrupted 2-0 Vicryl sutures and the dermis with interrupted 3-0 Vicryl sutures. The skin is closed with a running 4-0 subcuticular Monocryl and steri-strips. The patient is discharged from the recovery room with standard post-op follow-up.

## Results

The described surgical technique was performed in three patients who failed conservative management. The first patient was a 70-year-old female with a 3-year history of left buttock and leg pain. She underwent multiple lumbar epidural injection and two lumbar foraminotomies without any relief. She was found to have a trigger point in the region overlying the left piriformis musculature and sciatic nerve. She did not undergo EMG studies of her lower extremities at our facility. She failed conservative management with physical therapy and 2 trigger point injections with only temporary resolution of her symptoms. Given the resolution of her symptoms with trigger point injections, the decision was made to proceed with surgery.

The second patient was a 51-year-old female with a 1-year progressive history of right buttock pain that radiated down the right leg. Her lumbar MRI was negative, and she was found to have classic symptoms of PS including a trigger point at the piriformis musculature overlying the sciatic nerve on the right. She failed conservative management of physical therapy, medications, and trigger point injections. Her EMG findings were inconclusive. Given her continued pain and failure of conservative management, the decision was made to proceed with surgery.

The third patient was a 39-year-old female with a multi-year history of pain that started in her right buttocks and radiated down her right leg. She was diagnosed with PS and had a classic trigger point at the piriformis musculature overlying the sciatic nerve on the right. She failed conservative management with physical therapy and trigger point injections with only temporary relief. Her EMG results were inconclusive. However, given her temporary relief with the trigger point injections, the decision was made to proceed with surgery.

All of our patients had immediate relief of their symptoms after surgery. Our median length of follow-up was 12 months with a range of 6–18 months postoperative. All 3 patients returned to clinic for their scheduled 2-week postoperative exam and incision check. Additionally, they were all seen in clinic at 3 months and 6 months postoperatively. One patient moved after her 6-month follow-up, and the last 2 were seen at 12 months and 18 months, respectively. At their last follow-up, 2 out of the 3 patients were still completely symptom free with the third patient reporting pain associated with lumbar radiculopathy at her 18-month follow-up She was symptom free for 12 months following her surgery but developed a new pain in her left leg that she stated was different from her initial sciatic pain. This new pain was successfully treated with conservative management. There were no surgical complications with any of the patients.

## Discussion

PS is a controversial condition in which the sciatic nerve is compressed by the piriformis muscle causing buttock and leg pain. Patients typically have a classic trigger point in the region of the piriformis muscle overlying the sciatic nerve [7, 8]. PS accounts for approximately 0.3–0.6% of all cases of sciatic nerve pain with a reported female to male ratio of 6:1 [9]. The condition has initially treated by conservative management including physical therapy, medications, and localized injections [5, 10, 11]. Surgical management has been reserved for refractory cases of PS that do not respond to conservative treatment. The two most documented treatment methods are open vs endoscopic decompression of the sciatic nerve by the release of the piriformis muscle [1].

Traditional open surgical treatment of PS consists of a long curvilinear incision in the shape of a reverse question mark around the entire lateral aspect of the gluteus maximus with an inferior stem at the base of the gluteus into the midline of the hamstring muscle. The gluteal muscle is then divided and reflected medial to expose the sciatic nerve. The nerve can then be traced up to the sciatic notch, relieving any constrictions on the way. The open method is a beneficial approach because it allows full exposure of the sciatic nerve and important vasculature in the area [1, 2, 8, 12]. However, the pitfalls of the open surgical approach are very large incisions with increased morbidity. There is an increased risk of bleeding, scaring, and potential for recurrence as compared to a more minimally invasive approach. Additionally, there is an increased recovery time and prolonged hospitalizations in patients that undergo open surgical management as compared to minimally invasive techniques [1].

Endoscopic surgical treatment of PS consists of the placement of two ports in the thigh using the femur as the primary landmark. The first port is placed 1 cm superior to the tip of the greater trochanter, while the second port is placed approximately 6–7 cm distal from the first. The ports are placed through small stab incisions in the skin. The sciatic nerve is then identified, and any constrictions are released with the use of an endoscope [6, 13]. The benefits of this treatment method are that it allows for a minimally invasive surgical approach, which leads to a faster recovery and shorter hospital stay. Additionally, it has decreased morbidity as compared to the open procedure [14]. The significant limitation of the endoscopic approach is the very steep learning curve in the use of endoscopic equipment and arthroscopy before attempting this procedure. Also, the approach provides significantly decreased visualization as compared to the open procedure and carries a higher risk of injury to local vasculature and nerves [1, 14].

As an alternative to the above procedures, the minimally invasive approach described in this manuscript provides the advantages seen in both the traditional open and endoscopic methods. Our surgical approach provides excellent visualization of critical structures with minimal morbidity and short hospitalization. With the small incision and direct corridor to the nerve constriction, this minimally invasive approach can be mastered without requiring the steep learning curve as seen in the endoscopic approach. Most importantly, bleeding and injury to surrounding structures are less likely and easier to control as compared to the endoscopic method. Additionally, all three of our surgical procedures using this technique were performed as outpatient procedures with long-term pain relief without prolonged hospital stay and increased morbidity as seen in the traditional open surgical technique.

The primary limitation of our study is the small sample size with only 3 patients treated, and they were all women. Given that piriformis syndrome does have a 6:1 female to male ratio, it is not surprising that with such a small sample size, all outpatients were women. The small sample size of our study is due to the low incidence of piriformis syndrome already mentioned and the even lower incidence of patient’s who require surgical intervention, which should be reserved for medically refractory cases [9]. In addition, the follow-up is relatively short with a median follow-up of 1 year. Finally, the procedure was assessed at only one institution by a single attending surgeon.

The primary limitation of this surgical technique is that it requires neuromonitoring in order to carry it out correctly. Neuromonitoring can be an expensive addition to surgery and is not always readily available. Additionally, only clinicians that have a basic understanding of peripheral nerves and gluteal anatomy should perform this surgical technique.

## Conclusion

This minimally invasive technique for treating piriformis syndrome is an effective treatment for this patient population. The positive results seen in our patients utilizing the novel integration of pre-operative trigger point localization coupled with intraoperative neuromonitoring make this surgical approach an attractive option.

## Data Availability

Data sharing not applicable to this article as no datasets were generated or analyzed during the current study.

## Abbreviations

PS: Piriformis syndrome
MRI: Magnetic resonance imaging
EMG: Electromyography
